# TNFα Blockade Inhibits Both Initial and Continued Control of Pulmonary *Coccidioides*

**DOI:** 10.3389/fcimb.2021.796114

**Published:** 2022-01-31

**Authors:** Daniel A. Powell, Lisa F. Shubitz, Christine D. Butkiewicz, Hien T. Trinh, Fariba M. Donovan, Jeffrey A. Frelinger, John N. Galgiani

**Affiliations:** ^1^ Valley Fever Center for Excellence, University of Arizona, Tucson, AZ, United States; ^2^ Department of Immunobiology, University of Arizona, Tucson, AZ, United States; ^3^ Department of Medicine, University of Arizona, Tucson, AZ, United States

**Keywords:** *Coccidioides*, valley fever, TNFα, biological response modifiers, pathogenesis

## Abstract

Tumor necrosis factor alpha (TNFα) is a pluripotent cytokine that is important in many infections, though its role in *Coccidioides* infection remains poorly understood. The need to understand TNFα in *Coccidioides* infection has increased recently with the widespread use of TNFα inhibitors for a wide variety of autoimmune conditions. Here, we couple the newly developed *Coccidioides* infection model using strain Cp1038 and C57BL/6 × DBA/2J F1 (B6D2F1) mice. B6D2F1 mice develop long-lasting control of Cp1038. Treatment of B6D2F1 mice with anti-TNFα antibodies permits significant fungal proliferation and death. Additionally, we show that antibody treatment limited to the first 2 weeks of infection was sufficient to induce this same loss of fungal control. Importantly, anti-TNFα antibody treatment initiated after fungal control leads to a loss of host control. These results highlight the importance of TNFα in both the initial control of murine *Coccidioides* and ongoing suppression of the fungal disease.

## Introduction

Coccidioidomycosis (CM) is caused by the soil-inhabiting dimorphic fungi *Coccidioides immitis* and *Coccidioides posadasii* found in endemic regions of the Southwestern United States, Mexico, and other parts of the Western Hemisphere (Nguyen et al., 2013; Galgiani et al., 2016; Freedman et al., 2018). Despite the relatively limited geographic distribution, at least 150,000 infections occur annually and estimates of its economic impact for Arizona and California total $1.4 billion/year. Most infections are self-limited but can cause illness lasting for months. In approximately 1% of all CM infections, extrapulmonary dissemination occurs, leading to progressive and destructive lesions in other organs, a complication known as disseminated CM (DCM) (Galgiani et al., 2016). DCM is much more likely in patients with broadly suppressed cellular immunity either from comorbidities or immunosuppressive therapy (Nguyen et al., 2013; Odio et al., 2017).

In the past few years, the use of biological response modifiers (BRMs) has greatly expanded. Among the first and most commonly used are tumor necrosis factor alpha (TNFα) inhibitor therapies: infliximab (Remicade), adalimumab (Humira), golimumab (Simponi), and etanercept (Enbrel) (Meroni et al., 2015; Komaki et al., 2017; Gerriets et al., 2021). All of these drugs have been reported to be associated with severe *Coccidioides* infection, but not all patients treated with these drugs who become infected with *Coccidioides* experience severe disease (Blair et al., 2019).

Our previous characterization of Cp1038 highlighted two key developments leading to these studies (Shubitz et al., 2021). Initially, we observed that mice deficient in TNFα were much more susceptible to intranasal infection with Cp1038 when compared to wild-type (WT) B6 mice, highlighting a critical role for TNFα in the murine infection model. Additionally, we found that in pulmonary infections, the B6D2F1 mouse was highly resistant to infection with Cp1038. This F1 hybrid of C57BL/6 and DBA/2J mice when infected with Cp1038 develops a state of control with low lung fungal burdens, minimal dissemination, and no mortality over 16 weeks, the longest time point tested. This static infection state is similar to what is seen in the normal course of human infection. Coupled together, these insights led to a model for testing the effect of TNFα BRMs on *Coccidioides* infection as described here.

## Results

### TNFα Blockade Inhibits *Coccidioides* Resistance in B6D2F1 Mice

**Figure 1:**
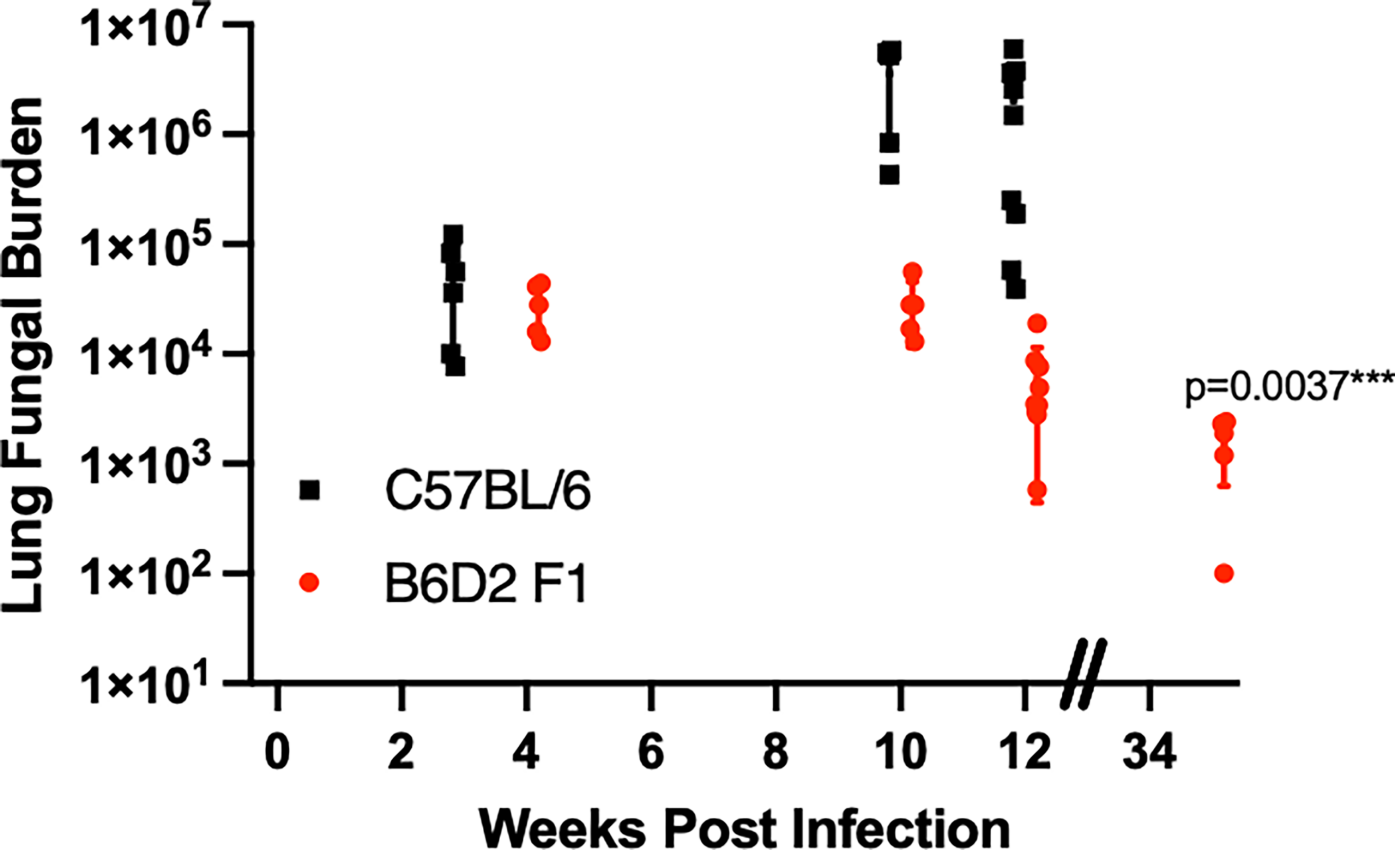
B6D2F1 mice have controlled lung fungal burdens after Cp1038 infection. C57BL/6 (black squares) or B6D2F1 (red circles) were intranasally infected with ~50 arthroconidia of Cp1038. At the indicated time points, 5–9 mice from each group were sacrificed, and lung fungal burden was determined. Data are represented as mean with standard deviation. B6D2F1 burdens were analyzed for differences compared to the initial 4-week burden using a Kruskal–Wallis on log-transformed data. Figure is combined from three separate infections with various planned end points.

We have previously shown that B6 mice deficient in TNFα succumb to Cp1038 infection much more rapidly than their normal B6 counterparts. All TNFα-deficient mice died by day 35 compared to deaths that began after day 55 in B6 mice (Shubitz et al., 2021). Additionally, we have shown that B6D2F1 mice are resistant to lethal infection with Cp1038. The infected B6D2F1 mice have a low fungal burden over time (Shubitz et al., 2021). To determine the kinetics of control of Cp1038, B6D2F1 mice were intranasally infected with Cp1038 and sacrificed at predetermined time points for lung fungal burden. The peak fungal burden was observed at 4 weeks post infection. Similar burdens were found at 10 and 12 weeks post infection. This is different from the lethal infections in C57BL/6J mice that show increasing fungal burdens 10 weeks after infection. A group of B6D2F1 sacrificed at 33 weeks (9 months) post infection showed a significantly (p = 0.0037) decreased fungal burden compared to 4 weeks **(** **Figure 1** **)**. Having demonstrated that the B6D2F1 mouse develops a long-lasting but low-level fungal burden, we sought to evaluate the effects of immunosuppression on this control.

**Figure 2:**
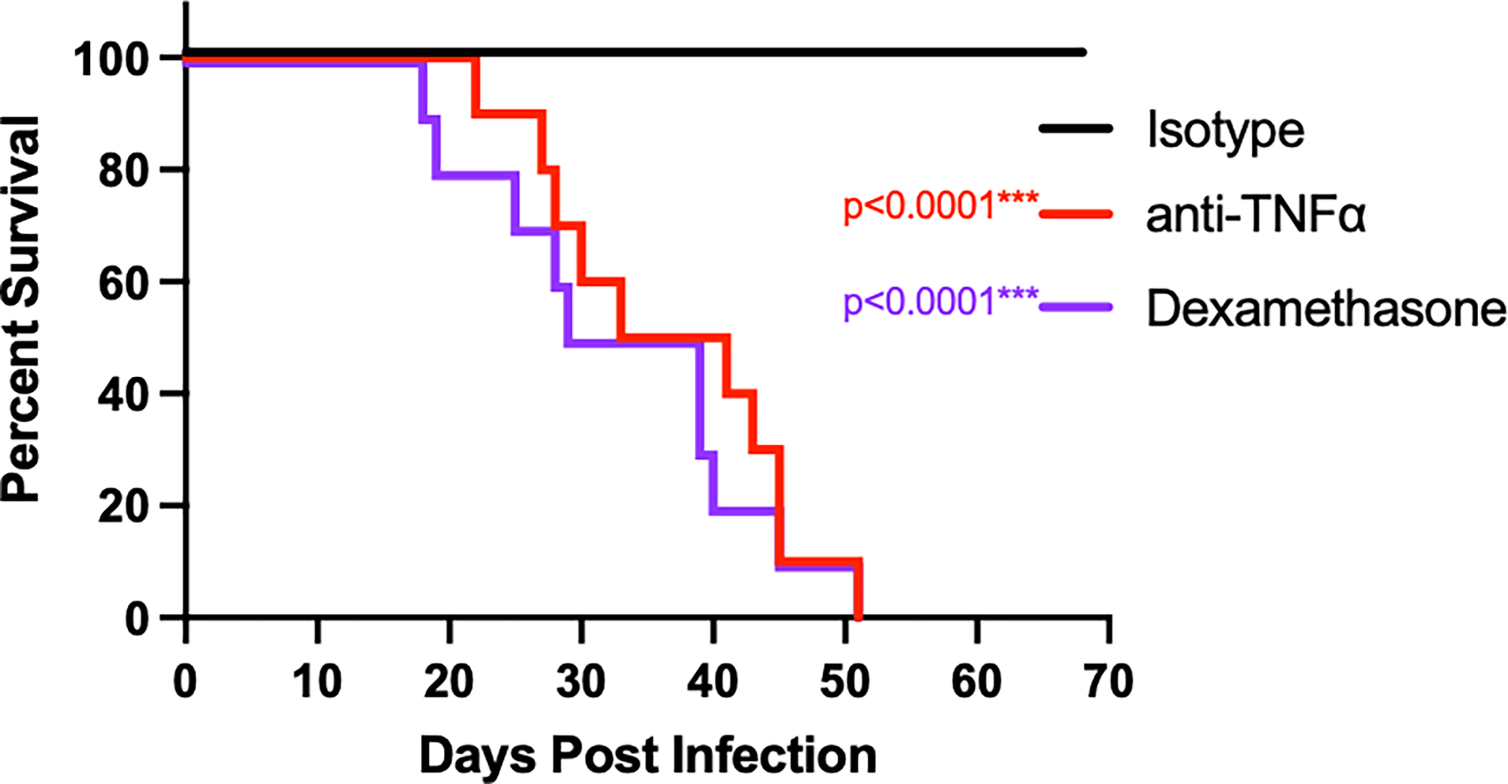
Treatment of B6D2F1 with anti-TNFα antibody results in progressive *Coccidioides* disease. B6D2F1 mice were treated with 500 μg isotype control (black line) or anti-TNFα antibody (red line) starting on day -2 before infection; dosing was continued on an alternating q3/4d schedule. An additional cohort of controls was treated with dexamethasone in their drinking water (purple line). On day 0, mice were infected with ~50 arthroconidia of Cp1038 and followed for disease progression.

To determine if TNFα is critical for establishing control, anti-TNFα monoclonal antibody was administered intraperitoneally (IP) to B6D2F1 mice starting 2 days prior to infection with Cp1038 and continued on an alternating q3/q4 schedule (Shen et al., 2019). For controls, mice were administered isotype-matched control antibody IP or the broadly immunosuppressive glucocorticoid dexamethasone in drinking water. Mice were followed for survival and disease progression (**Figure 2**). Mice treated with isotype control survived to the scheduled experimental end point and showed no signs of disease. Similar to B6 mice deficient in TNFα, B6D2F1 mice treated with anti-TNFα showed disease progression, with all mice succumbing to infection by day 51 (p < 0.0001). The kinetics of disease progression were similar between anti-TNFα antibody treatment group (median survival 37 days) and mice being broadly suppressed with dexamethasone (median survival 34 days). This study demonstrates a critical role for TNFα in the control of *Coccidioides* in the highly resistant B6D2F1.

### Interruption of TNFα Blockade After *Coccidioides* Infection Does Not Rescue B6D2F1 Mice

**Figure 3:**
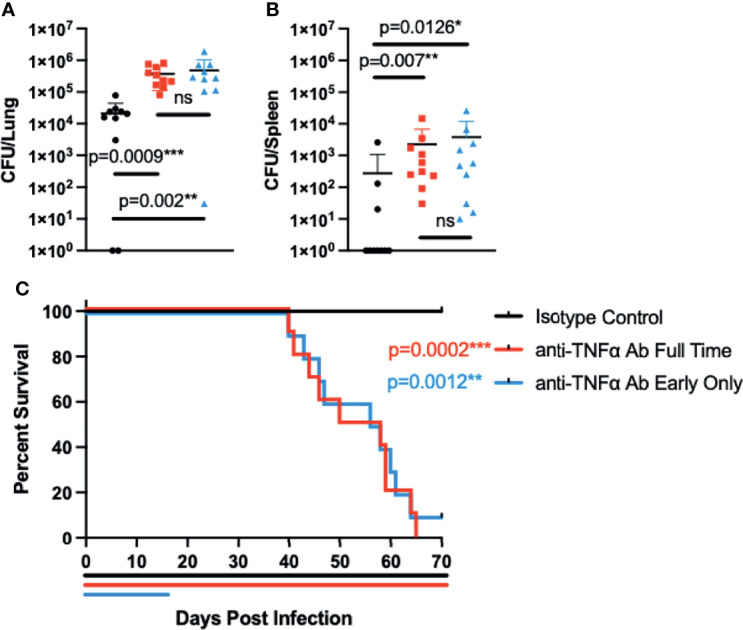
Interrupting treatment of B6D2F1 with anti-TNFα antibody after infection still results in progressive *Coccidioides* disease. B6D2F1 mice were treated with 500 μg isotype control (black symbols) or anti-TNFα antibody (red symbols) starting on day -2 before infection; dosing was continued on an alternating q3/4d schedule. A third group of B6D2F1 mice was only treated for the first 14 days of the infection (blue symbols). And 28 days after the infection, a subset of mice (n = 8/group) were sacrificed and lung **(A)** and spleen **(B)** fungal burdens were determined by serial dilution. The remaining mice (n = 10/group) were followed for disease progression **(C)**. Treatment timelines are indicated by colored lines below the survival graph. Data are represented as mean with standard deviation. Differences in fungal burden were tested for significance using a Kruskal–Wallis on log-transformed data. Survival differences were determined using Mantel–Cox. ns, not significant.

Having established that *Coccidioides* resistance can be overcome in B6D2F1 mice through TNFα blockade, we wanted to examine if halting of treatment after infection would allow for restoration of resistance. For these experiments, we treated groups of mice with control or anti-TNFα antibody starting 2 days before infection as above. In one subset, we halted the treatment at 14 days after infection; the remaining group received antibody throughout the experiment. Subsets from all treatment groups were sacrificed 28 days after infection to determine intermediate fungal burdens in the lung and spleen **(** **Figures 3A, B** **)**. Remaining mice were monitored for overall disease progression **(** **Figure 3C** **)**. As expected, mice receiving continuous anti-TNFα antibody treatment had significantly higher lung fungal burdens than isotype-treated controls (p = 0.0009). Spleen fungal burdens in continuous anti-TNFα antibody-treated mice were also increased compared to isotype-treated controls (p = 0.007), indicating increased dissemination outside of the thoracic cavity. However, mice that had treatment stopped also showed increased fungal burdens compared to the isotype-treated groups in both the lungs and spleens (p = 0.002 and p = 0.0126), and there was no difference between the fungal burdens among animals treated for 2 weeks or the entire length of the experiment **(** **Figures 3A, B** **)**. When following disease progression, the isotype-treated animals survived throughout the planned length of the experiment (70 days). Mice treated for the length of the experiment showed progressive disease and had a mean time to death of 54 days. Of note, mice treated for only the initial 2 weeks of the experiment also had progressive disease with a mean time to death of 57 days, similar to mice being treated the entire experiment **(** **Figure 3C** **)**.

### TNFα Blockade Disrupts Stable Control of *Coccidioides* Infection in B6D2F1 Mice

**Figure 4:**
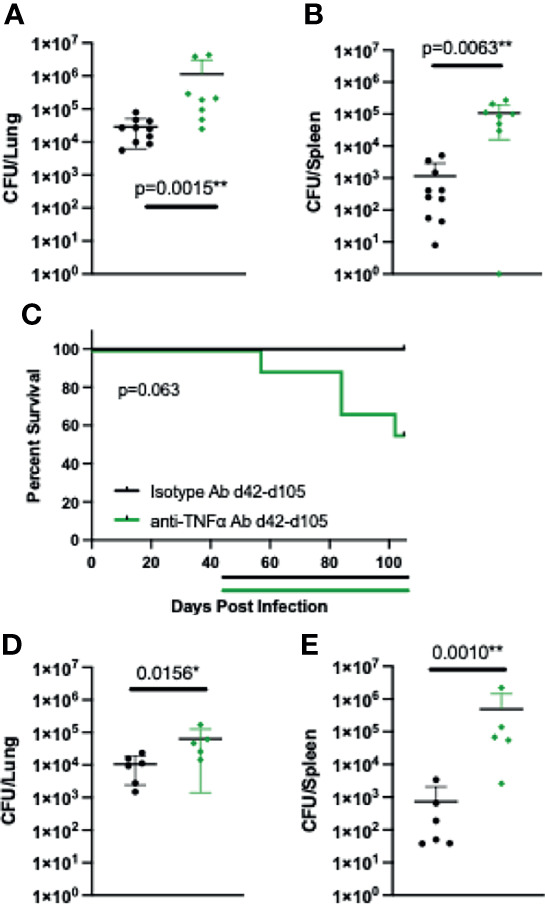
Starting treatment of B6D2F1 with anti-TNFα antibody after infection is controlled still results in progressive *Coccidioides* disease. B6D2F1 mice were treated with 500 μg isotype control (black symbols) or anti-TNFα antibody (green symbols) starting on day 42 after infection with ~50 arthroconidia of Cp1038; dosing was continued on an alternating q3/4d schedule. And 28 days after the start of anti-TNFα therapy, a subset of mice (n = 8/group) was sacrificed, and lung **(A)** and spleen **(B)** fungal burdens were determined by serial dilution. The remaining mice (n = 10/group) were followed for disease progression **(C)**. Treatment timelines are indicated by colored lines below the survival graph. Data are represented as mean with standard deviation. At the planned end of the experiment (105 days), surviving mice were sacrificed and lung **(D)** and spleen **(E)** fungal burdens were determined by serial dilution. Differences in fungal burden were tested for significance using a Kruskal–Wallis on log-transformed data. Survival differences were determined using Mantel–Cox.

To determine if TNFα has a role in the long-term maintenance of control of *Coccidioides* in B6D2F1 mice, mice were intranasally infected with Cp1038 and allowed to establish stable control of the infection as seen previously. Starting 42 days after infection, mice were treated with control or anti-TNFα antibody on the same q3/q4 schedule as above. Twenty-eight days after starting therapy, groups of mice were sacrificed for lung and spleen fungal burdens **(** **Figures 4A, B** **)**. Mice treated with anti-TNFα showed an increased fungal burden in the lungs and spleen compared to control-treated animals (p = 0.0015, p = 0.0062), highlighting a role for TNFα in the long-term maintenance of *Coccidioides* infection in B6D2F1 mice. A second cohort of mice was followed for survival and disease progression up to 105 days post infection (42 days after starting anti-TNFα antibody treatment). Mice with stable Cp1038 infection treated with anti-TNFα antibody began to die 2 weeks after the treatment was initiated **(** **Figure 4C** **)**, and the lungs and spleens showed grossly severe disease not present in the isotype-treated controls. At the planned end of the experiment (105 days), the surviving mice were sacrificed for lung and spleen fungal burden **(** **Figures 4D, E** **)**. While the survival curves were not significantly different (p = 0.063), they are divergent. Of the surviving mice, the anti-TNFα antibody-treated group had a slightly elevated lung fungal burden compared to isotype-treated animals (p = 0.0156). Quantitative plating of the spleen showed that treated mice have significantly more dissemination (p = 0.0010). As expected, there was little change in the isotype-treated burdens between 28 and 105 days post infection. Together, these data show a role for TNFα not only in establishing control but also in long-term control of the *Coccidioides* infection, particularly in relation to disseminated disease.

## Discussion

The use of TNF inhibitors is accompanied by a potential risk of fungal infection, leading to a black box warning related to potential opportunistic infections (Taroumian et al., 2012; Dobler, 2016; Ogawa et al., 2020). Little in-depth information on *Coccidioides* and TNF inhibitors exists, mostly limited to case reports and retrospective studies (Blair et al., 2019). While small, these retrospective studies show that screening of patients starting TNF inhibitors for *Coccidioides* can be beneficial in preventing severe disease. The exact risk factors that differentiate which patients being treated with TNF inhibitors will develop severe *Coccidiodes* disease are unclear. Development of our chronically infected B6D2F1 mouse model coupled with anti-TNFα antibodies allows us to interrogate the changes in disease progression after this BRM treatment (Shubitz et al., 2021).

The B6D2F1 mouse, when infected with Cp1038, develops a long-lasting, chronic disease state with low fungal burdens in the lungs and spleens and no mortality. This is similar to the disease course seen in many human patients. This long-term control of infection is not seen in B6 mice that succumb to infection (Shubitz et al., 2021), adding the genetic variation with the B6D2F1 highlights there is a host genetic factor in control of disease. Initiating anti-TNFα before the infection results in lethal infection with a mean time to death of 34 days. Treatment with the broadly immunosuppressive dexamethasone leads to a similar time to death, indicating that TNFα is a critical factor in establishing control of *Coccidioides* in our infection model. Whether other targeted immunosuppressives such as Janus Kinase (JAK) inhibitors, Interlukin (IL)-1 receptor antagonists, or anti-Cluster of Differentiation 20 (CD20) antibodies have a similar effect remains to be explored but is possible in this newly developed model.

We also examined if a shorter treatment with the anti-TNFα antibodies after infection had a diminished effect on the disease progression. Surprisingly, stopping the treatment 2 weeks after infection had no alteration on the lethal disease. Two weeks after stopping treatment, there was increased fungal burden in mice that were previously treated with anti-TNFα antibody compared to untreated mice. There was no difference in the fungal burdens compared to mice still receiving anti-TNFα antibody, indicating that an early TNFα-dependent event establishes initial control of *Coccidioides* infection. Animals that had their treatment halted succumbed to infection in the same time period as mice still on anti-TNFα therapy. This similar time to death, even with the termination of anti-TNFα treatment, indicates that failure to develop a proper immune response to the initial infection has long-reaching consequences.

Not only does TNFα play a role in controlling the initial infection with *Coccidioides*, we demonstrated a continuing role in mice with established control of infection. Mice were infected for 42 days, allowing them to establish control of the infection. These mice were then treated with either anti-TNFα or isotype control antibody. Twenty-eight days after starting the anti-TNFα antibody, mice had significantly increased lung and spleen fungal burdens compared to the controls, indicating that these animals were starting to lose control of the fungal infection. Mice receiving anti-TNFα antibody also started to succumb to progressive disease. While both initial control and continued control are TNFα-dependent, whether the mechanisms are the same remains to be explored. Similar dependence on TNFα to maintain control of a pathogen has also been observed in other infections, including *Mycobacteria* (Mohan et al., 2001; Wallis et al., 2004; Deepe, 2005; Deepe et al., 2005; Rychly and DiPiro, 2005). This dependence has been linked to both innate cellular production of TNFα and adaptive production by T cells. In this model, there may be roles for both innate and adaptive TNFα, particularly as we see effects both early and late in infection.

Using our newly developed model, we have demonstrated that we can examine the risk of BRMs in *Coccidioides* infection. We have shown that blockade of the TNFα pathway causes a loss of control of *Coccidioides*. This loss of control happens even if treatment is stopped 2 weeks after the infection, even though animals do not succumb to infection for several more weeks. Disruption of TNFα after control has been established also leads to progressive disease. These findings taken together highlight the need for more careful study of BRMs and the potential risk in regions where fungal infections are endemic.

## Materials and Methods

### Fungal Strains

*C. posadasii* strain RMSCC1038 (Cp1038) was grown on 2× glucose yeast extract agar (GYE) (2% glucose, 1% yeast extract, and 1.5% agar) at 30°C for 12 weeks, and arthroconidia were harvested and enumerated using a hemacytometer as described previously (Shubitz et al., 2021). Cp1038 was filtered through Pellon Thermolam Plus to remove hyphal elements. Viability was determined by growth of 10-fold serial dilutions on 2× GYE at 37°C for 7 days. Animal inoculation suspensions were diluted in isotonic saline. All manipulations of live fungus were performed at biosafety level 3 and approved by the University of Arizona Institutional Biosafety Committee.

### Mice

Female C57BL/6J (Stock Number 000664) and B6D2F1/J (Stock Number 100006) were ordered at 6–8 weeks of age from The Jackson Laboratory. Mice were housed according to PHS standards; all procedures were approved by the Institutional Animal Care and Use Committee of the University of Arizona. Female mice were used for ease of housing and handling under biosafety level 3 conditions. We have historically seen no sex differences in *Coccidioides* infection. Mice were housed and manipulated in an animal biosafety level 3 facility for the duration of the infection studies.

### Infections and Immunosuppression

Groups of 5–10 mice were infected intranasally under ketamine-xylazine anesthesia with approximately 50 arthroconidia in 30 μl of isotonic sterile saline as previously described. For survival studies, mice were observed daily and euthanized if moribund [defined as >20% weight loss, inactivity and ruffled condition, or dehydration of 7%–10% based in skin turgor, or any central nervous system (CNS) signs]. Fungal burdens were quantified by serial dilution of homogenized tissue on 2× GYE at 37°C for 7 days, as described previously (Narra et al., 2016).

For antibody depletion, mice were injected subcutaneously with 500 μg of either anti-TNFα (CNTO5048 MP1B4.003) or isotype control (CNTO6601 MSCB149.001). For early or continued intervention, injections were started 2 days before infection and continued on an alternating q3/q4 schedule as described previously (Shen et al., 2019). Late treatment was started on day 42 after infection and continued on an alternating q3/q4 schedule. Both antibodies were gifts from Janssen Biotech.

Mice were given dexamethasone in drinking water, 6 mg/L, to administer approximately 1 mg/kg/day of dexamethasone (Yang and Healey, 1993).

### Statistical Analysis

Organ fungal burden data were log-transformed and analyzed using a Kruskal–Wallis analysis. Survival curves were analyzed by Mantel–Cox. Differences were considered significant at p < 0.05. All analyses were carried out using GraphPad Prism.

## Data Availability Statement

The raw data supporting the conclusions of this article will be made available by the authors without undue reservation.

## Ethics Statement

The animal study was reviewed and approved by the Institutional Animal Care and Use Committee of the University of Arizona.

## Author Contributions

DP wrote the initial article, designed the experiments, and collected and analyzed data. LS, CB, HT, and FD collected and analyzed data. JF and JG designed the experiments and analyzed data. All authors contributed to final editing of the article. All authors contributed to the article and approved the submitted version.

## Funding

This work was supported by a grant from the National Institutes of Health to JG (R01AI132140).

## Conflict of Interest

The authors declare that the research was conducted in the absence of any commercial or financial relationships that could be construed as a potential conflict of interest.

## Publisher’s Note

All claims expressed in this article are solely those of the authors and do not necessarily represent those of their affiliated organizations, or those of the publisher, the editors and the reviewers. Any product that may be evaluated in this article, or claim that may be made by its manufacturer, is not guaranteed or endorsed by the publisher.
